# The Association Between Physical Activity and Insulin Level Under Different Levels of Lipid Indices and Serum Uric Acid

**DOI:** 10.3389/fphys.2022.809669

**Published:** 2022-02-02

**Authors:** Yajuan Lin, Rui Fan, Zhujing Hao, Jiatian Li, Xiaolei Yang, Ying Zhang, Yunlong Xia

**Affiliations:** Department of Cardiology, First Affiliated Hospital of Dalian Medical University, Dalian, China

**Keywords:** physical activity, insulin, NHANES, SUA, lipids

## Abstract

**Objectives:**

Insulin resistance (IR) has been shown to play important role in the pathogenesis of type 2 diabetes mellitus (T2DM). There is an intricate interplay between IR, dyslipidemia, and serum uric acid (SUA) in people with and without diabetes. Physical activity has a positive impact on insulin sensitivity in insulin-resistant populations. However, the effect of different intensities of physical activity on insulin levels under different lipid indices and SUA levels is unclear.

**Methods:**

To explore the association between physical activity and insulin, we enrolled 12,982 participants aged above 18 years from the National Health and Nutrition Examination Survey (NHANES) conducted between 2009 and 2018. Next, we conducted multivariate logistic regression analyses, generated fitted smoothing curves, and visualized the data using generalized additive models.

**Results:**

Increased intensities of physical activity can significantly reduce insulin levels. The association between physical activity and insulin persisted even after adjusting for confounding factors, with *β* value (95% CI) = −17.10 (−21.64, −12.56) in moderate group, *β* value (95% CI) = −28.60 (−33.08, −24.11) in high group, respectively. High-intensity physical activity significantly lowered insulin levels in the lower and higher SUA tertiles, and three tertiles of LDL-c, HDL-c, and TG. Moreover, the link between physical activity and insulin was stronger in male individuals.

**Conclusion:**

This study shows that physical activity can significantly lower insulin levels, and high-intensity physical activity still has additional potential benefits for insulin levels, even in the condition of dyslipidemia and hyperuricemia.

## Introduction

Insulin resistance (IR) is defined as an impaired biologic response to glucose disposal and insulin stimulation of target tissues (mainly the liver, muscle, and adipose tissue), leading to a compensatory increase in beta-cell insulin production and hyperinsulinemia. IR can result in a series of metabolic consequences, such as dyslipidemia, hyperglycemia, visceral adiposity, hyperuricemia, hypertension, endothelial dysfunction, a prothrombotic state, and elevated inflammatory response (Freeman and Pennings, 2021). Fasting hyperinsulinemia was observed to precede the development of other characteristics of the syndrome, such as hypertension, hypertriglyceridemia, and low high-density lipoprotein cholesterol (HDL-c) by Haffner et al. (1992). Barnard et al. (1988) fed mice a high-fat sucrose diet for a few weeks and found that they developed skeletal muscle insulin resistance and hyperinsulinemia before mice developed hypertriglyceridemia, and hypertension, which resulted in MS. It is worth noting that IR is a key metabolic feature of obesity, type 2 diabetes mellitus (T2DM), and even type 1 diabetes mellitus (T1DM), and is associated with an increased risk of micro- and macro-vascular problems. It is increasingly becoming apparent that, even in the absence of diabetes, there is a link between IR and cardiovascular disease (CVD; Howard et al., 2000). Therefore, improving insulin sensitivity is an important part of preventing insulin resistance, diabetes, and CVD.

Physical activity (PA) improves insulin sensitivity in both normal and insulin-resistant people. Evidence suggests that insulin resistance (i.e., low insulin sensitivity) is the major underpinning link between physical inactivity and MS (Roberts et al., 2013). Thus, PA has been proposed for treating diabetes mellitus (Aune et al., 2015). Several recent epidemiological studies have found a link between PA and the occurrence of T2DM (Eriksson and Lindgarde, 1991; Manson et al., 1991). Through numerous changes in glucose transport and metabolism, PA enhances the effect of exercise on insulin sensitivity (Borghouts and Keizer, 2000). Therefore, aerobic exercise is generally considered the most adequate mode of exercise for improving insulin sensitivity.

It is well known that, in addition to controlling blood glucose levels, insulin regulates lipid metabolism by promoting lipid synthesis in the liver and fat cells while inhibiting lipolysis. Notably, PA may cause beneficial changes in lipid metabolism as well as improvements in hepatic glucose output regulation. In addition, the relationship between serum uric acid (SUA) and glucose/insulin balance is complicated. Based on pathophysiological and metabolic studies, hyperuricemia and IR are likely to interact. Hyperuricemia plays a role in the pathogenesis of T2DM, and insulin resistance by increasing inflammation recreation and oxidative stress (Lanaspa et al., 2012; Wan et al., 2016). IR, on the other hand, decreases uric acid excretion by increasing renal tubular sodium reabsorption and so producing hyperuricemia (Ter Maaten et al., 1997). Longitudinal studies on this issue have yielded inconsistent results. Increased uric acid levels have been linked to an increased risk of IR in several studies (Krishnan et al., 2012). Alternatively, IR might be a risk factor for later hyperuricemia on its own (Nakamura et al., 2014). According to the findings of these studies, the dynamic of the temporal relationship between hyperuricemia and IR is likely complex, as changes in one may precede those in the other.

Indeed, multiple studies in recent decades have demonstrated that increasing PA and cardiorespiratory fitness has a positive impact on each of the metabolic syndrome components (high waist circumference, dyslipidemia, hypertension, and insulin resistance; Duncan, 2006; Church, 2011; Zhang et al., 2017). However, there are currently no relevant studies evaluating the effect of PA on insulin levels under different levels of lipid indices (TG, LDL-c, and HDL-c) and SUA. This study explored the association between PA and insulin under different levels of lipid indices and SUA using a representative sample from the National Health and Nutrition Examination Survey (NHANES).

## Materials and Methods

### Study Population

The National Health and Nutrition Examination Study (NHANES), which is a representative survey of the national population in the United States, was conducted by the Centers for Disease Control and Prevention (CDC). Using a complicated, multistage, and probabilistic sampling approach, this study provides a wealth of information about the nutrition and health of the overall US population (Curtin et al., 2013; Patel et al., 2016). This cross-sectional study analyzed the data collected from 2009 to 2018, representing five cycles of the NHANES.^1^ The research ethical review board of the National Center for Health Statistics authorized the study procedures. A total of 12,982 participants over the age of 18 were included in the analysis after excluding participants under the age of 18 (*n* = 19,341), those with missing PA data (*n* = 69), and those with missing insulin (*n* = 14,183) and other key data (*n* = 3,119). Notably, signed informed consent had been obtained from each participant during data collection.

### Exposure Variables and Outcomes

The physical activity (the exposure variable) of participants between 2009 and 2018 was based on the Global Physical Activity Questionnaire (GPAQ; Hallal et al., 2012), which includes questions related to daily activities, leisure time activities, and sedentary activities. PA was then categorized into three levels (low, moderate, and high) according to the suggested MET score (Ainsworth et al., 2011).^1^

Insulin, the outcome variable, was measured by human insulin immunoassay using ROCHE ELECSYS 2010 at the Fairview-University Medical Center University Campus Collaborative Studies Clinical Laboratory Minneapolis, Minnesota between 2009 and 2012. The immunoenzymometric assay TOSOH AIA-900 Chemistry Analyzer was then used to measure insulin between 2013 and 2018 at the University of Missouri Columbia. Extensive quality control processes were performed by the analytical laboratory. External calibration was performed using whole-blood resources from the National Institute of Standards and Technology. The detailed information on PA and insulin is publicly available at http://www.cdc.gov/nchs/nhanes/.

### Covariates

Sex, smoking, PA, race/ethnicity, alcohol consumption, history of hypertension, and diabetes mellitus were collected as categorical variables, whereas age, body mass index (BMI), systolic blood pressure (SBP), waist circumference (WC), diastolic blood pressure (DBP), total cholesterol (TC), creatinine, aspartate aminotransferase (AST), and alanine aminotransferase (ALT) were collected as continuous variables. Detailed insulin and covariate information can be found at http://www.cdc.gov/nchs/nhanes/. The primary outcome was to determine the association between PA and insulin levels. Therefore, the results of the adjusted potential confounders model analyses were presented based on the recommendations of the STROBE statement (von Elm et al., 2007), when the covariates were added to the model, the odds ratio of the match was changed by at least 10%, and this covariate needed to be adjusted (Vandenbroucke et al., 2007). Consequently, the fully adjusted model was built using the following variables: age, sex, race/ethnicity, BMI, SBP, HDL, LDL-C, TG, ALT, AST, creatinine, glucose, SUA, smoking, and alcohol consumption.

### Statistical Analysis

All statistical analyses were performed using Empower Stats 2.2^2^ and package R.^3^ According to the NHANES protocol, all of the data were integrated into a single dataset, and data analysis took into account the masked variance and applied the suggested weighting methodology. Participants were divided into three groups based on the intensity of PA. To explore the differences among groups, the weighted *χ*^2^ test was utilized for categorical variables expressed as percentages, whereas the weighted linear regression model was applied for continuous variables expressed as the means ± SD. In the association analyses, a weighted multivariate logistic regression model was used to explore the relationship between PA and insulin. Insulin levels regression coefficients (*β* value) and 95% confidence intervals (CIs) were evaluated by constructing a series of hierarchical models that adjusted for potential confounders: Model I, adjusted for age, sex, and race; Model II, additionally adjusted for BMI, SBP, HDL-c, LDL-c, TG, ALT, AST, creatinine, glucose, SUA, smoking, and alcohol consumption. The weighted multivariate regression model also analyzed the association between the SUA, lipid indices (LDL-c, HDL-c, and TG), and PA (predictor), and insulin levels (outcome), and SUA, LDL-c, HDL-c, and TG were all analyzed as categorical variables and classified into three groups (tertiles). SUA (T1, T2, and T3), LDL-c (T1, T2, and T3), TG (T1, T2, and T3), and HDL-c (T1, T2, and T3) have different cut-off values indicated at the footnote of each table. Subgroup analyses were also performed based on sex. To further explain the association between PA (predictor) and insulin levels (outcome). The participants were classified into diabetes and non-diabetes subgroups according to clinical diagnoses. Sensitivity analysis was performed based on participants without diabetes status. Stratified analyses were conducted according to age (≤44, 45–59, ≥60), gender, race, LDL-c tertiles (T1, T2, and T3), HDL-c tertiles (T1, T2, and T3), TG tertiles (T1, T2, and T3), glucose tertiles (T1, T2, and T3), SUA tertiles (T1, T2, and T3), and BMI (<25, ≥25), and the obtained results were presented using a forest plot. The nonlinear link between SUA, LDL-c, HDL-c, TG, and insulin was further evaluated using smooth curve fits and generalized additive models. A value of *p* < 0.05 was considered statistically significant.

## Results

### Baseline Characteristics of the Participants

This study included 12,982 participants aged >18 years. The baseline demographic and clinical characteristics are shown in Table 1, with the weighted characteristics of the participant’s sub-classified based on PA (low, moderate, and high). Results showed that the mean values of HbA1c, glucose, BMI, WC, TG, creatinine, insulin, BUN, and SBP were significantly lower in the high-intensity PA group than in the other two groups. However, the mean values of HDL-c, AST, ALT, and DBP levels were significantly higher in the high-intensity PA group than in the other two groups. Participants in the high-intensity PA group were younger (*p* < 0.001) and had a higher proportion of males and alcohol consumption (*p* < 0.001) compared to the other two groups, but with a lower proportion of hypertension, diabetes, Non-Hispanic black, and smokers (*p* < 0.001).

**Table 1:** Basic characters.

Variable	Physical activity			*p* value
	Low (*n* = 3,289)	Moderate (*n* = 4,989)	High (*n* = 4,704)	
Age (years)	53.52 ± 17.69	49.64 ± 17.31	41.59 ± 15.47	<0.001
BMI (kg/m^2^)	30.47 ± 7.74	29.15 ± 7.20	28.16 ± 6.43	<0.001
WC (cm)	103.30 ± 17.51	100.05 ± 16.82	96.93 ± 15.90	<0.001
Sex				<0.001
Male	37.94	40.71	61.49	
Female	62.06	59.29	38.51	
Race (%)				<0.001
Mexican American	10.32	7.47	9.28	
Non-hispanic white	62.37	67.69	66.57	
Non-hispanic black	11.84	10.44	9.82	
Other race/ethnicity	15.47	14.40	14.33	
Alcohol consumption (%)				<0.001
Moderate alcohol use	53.97	64.04	68.83	
High alcohol use	0.92	1.11	1.52	
Nondrinker	41.70	31.45	23.66	
Smoking (%)	22.60	22.86	22.32	<0.001
Hypertension (%)	55.49	48.99	37.08	<0.001
SBP (mmHg)	131.31 ± 22.27	129.23 ± 21.50	125.73 ± 20.34	<0.001
DBP (mmHg)	67.59 ± 16.27	68.58 ± 15.55	69.80 ± 13.35	<0.001
Diabetes mellitus (%)	21.17	14.66	7.41	<0.001
Laboratory data				
HbA1c (%)	5.88 ± 1.13	5.69 ± 0.96	5.49 ± 0.78	<0.001
HDL-c (mmol/L)	1.38 ± 0.44	1.42 ± 0.41	1.42 ± 0.44	<0.001
TG (mmol/L)	1.57 ± 1.55	1.48 ± 1.04	1.36 ± 1.53	<0.001
ALT (IU/L)	23.89 ± 16.85	24.49 ± 16.98	25.81 ± 18.45	<0.001
AST (IU/L)	24.94 ± 19.14	24.50 ± 13.17	25.35 ± 18.81	0.042
BUN (mmol/L)	5.19 ± 2.50	4.81 ± 1.91	4.80 ± 1.56	<0.001
LDL-c (mmol/L)	2.89 ± 0.94	2.93 ± 0.91	2.92 ± 0.90	0.231
Creatinine (μmol/L)	79.86 ± 54.10	75.47 ± 34.22	77.83 ± 19.02	<0.001
Glucose (mmol/L)	5.95 ± 2.10	5.63 ± 1.68	5.35 ± 1.32	<0.001
SUA (μmol/L)	326.15 ± 87.57	320.51 ± 83.23	326.82 ± 81.02	<0.001
Insulin (pmol/L)	99.05 ± 116.98	81.95 ± 100.11	70.45 ± 83.63	<0.001

Mean ± SD for continuous variables: the value of *p* was calculated by the weighted linear regression model. (%) for categorical variables: The value of *p* was calculated by the weighted chi-square test. HbA1c, hemoglobin A1c; BMI, body mass index; WC, waist circumference; SBP, systolic blood pressure; DBP, diastolic blood pressure; ALT, alanine aminotransferase; AST, aspartate aminotransferase; TG, Triglyceride; HDL-c, high-density lipoprotein cholesterol; LDL-c, low-density lipoprotein cholesterol; SUA, serum uric acid; and BUN, blood urea nitrogen.

### The Association Between PA and the Level of Insulin (pmol/L)

**Figure 1:**
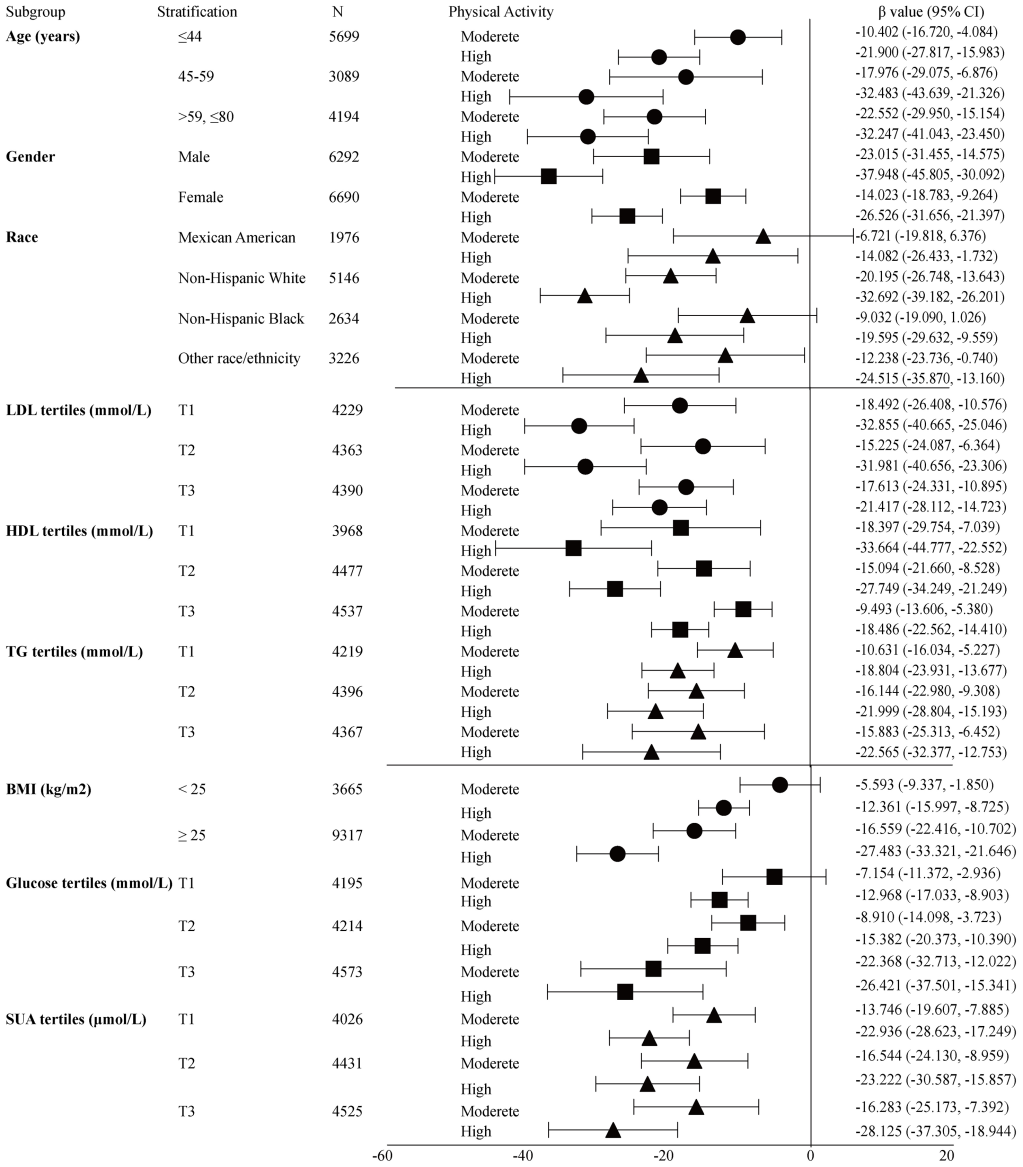
Crude subgroup analyses on effect of physical activity on insulin. Glucose tertiles = T1 1.05–4.94 mmol/L; T2 5.00–5.50 mmol/L; T3 5.55–24.81 mmol/L; HDL-c tertiles = T1 0.16–1.14 mmol/L; T2 1.16–1.47 mmol/L; T3 1.5–5.84 mmol/L; LDL-c tertiles = T1 0.23–2.43 mmol/L; T2 2.46–3.15 mmol/L; T3 3.18–9.69 mmol/L; TG tertiles = T1 0.10–0.89 mmol/L; T2 0.90–1.47 mmol/L; T3 1.48–68.38 mmol/L; SUA tertiles = male T1 23.80–321.20 μmol/L; T2 327.10–386.60 μmol/L; T3 392.60–773.20 μmol/L; Female T1 23.80–243.90 μmol/L; T2 249.80–303.30 μmol/L; and T3 309.30–1,070.60 μmol/L.

Table 2 shows the results of the multivariate regression analyses. In the unadjusted model, the high- and moderate-intensity PA groups were negatively correlated with insulin [*β* value (95% CI) = −17.10 (−21.64, −12.56), *β* value (95% CI) = −28.60 (−33.08, −24.11), respectively] compared to the low-intensity PA group. After adjusting for confounders, this association persisted in model II [*β* value (95% CI) = −8.74 (−12.90, −4.57), *β* value (95% CI) = −12.46 (−16.80, −8.11), respectively]. When grouped by sex, this association persisted in males, with *β* value (95% CI) = −15.15 (−22.72, −7.59), *β* value (95% CI) = −19.43 (−26.74, −12.13), respectively. However, in females, the significant negative association only persisted in the high-intensity PA group after adjusting for confounders (Model II), with *β* value of (95% CI) = −6.61 (−11.66, −1.56). Forest plot showed the crude subgroup analyses on the effect of PA on insulin (Figure 1). In the unadjusted analyses, negative associations were observed between PA and insulin in all stratified analyses.

**Table 2:** The association between physical activity and insulin.

Physical activity	Crude model		Model I		Model II	
	*β* (95% CI)	*p* value	*β* (95% CI)	*p* value	*β* (95% CI)	*p* value
Low	Ref.		Ref.		Ref.	
Moderate	−17.10 (−21.64, −12.56)	<0.001	−16.66 (−21.21, −12.10)	<0.001	−8.74 (−12.90, −4.57)	<0.001
High	−28.60 (−33.08, −24.11)	<0.001	−31.11 (−35.83, −26.39)	<0.001	−12.46 (−16.80, −8.11)	<0.001
**Male**						
Low	Ref.		Ref.		Ref.	
Moderate	−23.02 (−31.46, −14.58)	<0.001	−22.356 (−30.83, −13.89)	<0.001	−15.15 (−22.72, −7.59)	<0.001
High	−37.95 (−45.81, −30.09)	<0.001	−37.74 (−45.89, −29.59)	<0.001	−19.43 (−26.74, −12.13)	<0.001
**Female**						
Low	Ref.		Ref.		Ref.	
Moderate	−14.02 (−18.78, −9.26)	<0.001	−13.08 (−17.85, −8.32)	<0.001	−4.38 (−8.88, 0.13)	0.057
High	−26.53 (−31.66, −21.40)	<0.001	−25.38 (−30.68, −20.08)	<0.001	−6.61 (−11.66, −1.56)	0.010

Model I: age, sex, and race/ethnicity were adjusted. Model II: age, sex, race/ethnicity, BMI, SBP, HDL-c, LDL-c, TG, ALT, AST, SUA, glucose, creatinine, smoking, and alcohol consumption were adjusted.

### The Effect of PA and SUA, Lipid Indices (LDL-c, HDL-c, and TG) Interaction on the Insulin Levels

#### The Effect of PA on Insulin Based on SUA Tertiles

Supplementary Figure S1A shows that there was a positive correlation between SUA level and insulin, and the level of insulin decreased as the intensity of PA improved under the same SUA level (Supplementary Figure S1B). Table 3 shows the interactive analyses between SUA and PA on the level of insulin. In the lower SUA tertile, significant differences were only observed in the high-intensity PA group, with *β* values (95% CI) = −9.75 (−15.33, −4.16) adjusted *β* values. However, in the upper SUA tertile, the adjusted *β* values (95% CI) were −13.99 (−21.67, −6.31) and −18.883 (−27.42, −10.34), respectively. The negative association persisted in males, with an adjusted *β* value (95% CI) = −15.10 (−25.19, −5.01) in the lower SUA tertile, and *β* values (95% CI) = −29.14 (−43.68, −14.59), −29.48 (−43.83, −15.12) in the upper SUA tertile. However, in females, considerable differences were only observed in the high-intensity PA group in the lower SUA tertile [*β* values (95% CI) = −6.18 (−11.88, −0.47), *p* = 0.034; Supplementary Table S1].

**Table 3:** The association between physical activity and insulin grouped by SUA, LDL-c, HDL-c, and TG tertiles.

Physical activity	Tertile 1 of SUA		Tertile 2 of SUA		Tertile 3 of SUA	
	***β* (95% CI)**	***p* value**	***β* (95% CI)**	***p* value**	***β* (95% CI)**	***p* value**
Low	Ref.		Ref.		Ref.	
Moderate	−2.87 (−8.39, 2.66)	0.309	−6.37 (−14.11, 1.38)	0.107	−13.99 (−21.67, −6.31)	<0.001
High	−9.75 (−15.33, −4.16)	<0.001	−6.79 (−14.68, 1.10)	0.092	−18.88 (−27.42, −10.34)	<0.001
**Male**						
Low	Ref.		Ref.		Ref.	
Moderate	−2.66 (−13.23, 7.92)	0.622	−8.62 (−21.82, 4.59)	0.201	−29.14 (−43.68, −14.59)	<0.001
High	−15.10 (−25.19, −5.01)	0.003	−8.58 (−21.29, 4.14)	0.186	−29.48 (−43.83, −15.12)	<0.001
**Physical activity**	**Tertile 1 of LDL-c**		**Tertile 2 of LDL-c**		**Tertile 3 of LDL-c**	
	***β*** **(95% CI)**	***p*** **value**	***β*** **(95% CI)**	***p*** **value**	***β*** **(95% CI)**	***p*** **value**
Low	Ref.		Ref.		Ref.	
Moderate	−11.16 (−19.14, −3.18)	0.006	−6.15 (−14.99, 2.70)	0.173	−10.29 (−17.09, −3.49)	0.003
High	−9.90 (−18.37, −1.43)	0.022	−16.21 (−25.32, −7.10)	<0.001	−15.26 (−22.33, −8.18)	<0.001
**Male**						
Low	Ref.		Ref.		Ref.	
Moderate	−25.45 (−40.23, −10.66)	<0.001	−4.09 (−19.83, 11.66)	0.611	−13.56 (−27.10, −0.02)	0.049
High	−22.75 (−37.80, −7.69)	0.003	−22.37 (−37.32, −7.43)	0.003	−17.27 (−29.99, −4.53)	0.008
**Physical activity**	**Tertile 1 of HDL-c**		**Tertile 2 of HDL-c**		**Tertile 3 of HDL-c**	
	***β*** **(95% CI)**	***p*** **value**	***β*** **(95% CI)**	***p*** **value**	***β*** **(95% CI)**	***p*** **value**
Low	Ref.		Ref.		Ref.	
Moderate	−20.85 (−31.67, −10.02)	<0.001	−5.15 (−11.40, 1.10)	0.107	−3.09 (−7.12, 0.94)	0.133
High	−24.19 (−35.29, −13.08)	<0.001	−10.33 (−16.96, −3.70)	0.002	−6.20 (−10.39, −2.01)	0.004
**Male**						
Low	Ref.		Ref.		Ref.	
Moderate	−26.98 (−41.79, −12.18)	<0.001	−6.48 (−15.27, 2.31)	0.148	1.23 (−7.67, 10.13)	0.787
High	−28.39 (−42.75, −14.02)	<0.001	−12.55 (−21.03, −4.06)	0.004	−4.25 (−12.80, 4.30)	0.330
**Physical activity**	**Tertile 1 of TG**		**Tertile 2 of TG**		**Tertile 3 of TG**	
	***β*** **(95% CI)**	***p*** **value**	***β*** **(95% CI)**	***p*** **value**	***β*** **(95% CI)**	***p*** **value**
Low	Ref.		Ref.		Ref.	
Moderate	−6.75 (−11.78, −1.72)	0.009	−10.18 (−17.15, −3.21)	0.004	−8.41 (−17.03, 0.21)	0.056
High	−9.33 (−14.30, −4.36)	<0.001	−15.38 (−22.66, −8.09)	<0.001	−11.36 (−20.87, −1.84)	0.019
**Male**						
Low	Ref.		Ref.		Ref.	
Moderate	−11.26 (−21.40, −1.12)	0.030	−21.58 (−32.48, −10.67)	<0.001	−9.03 (−24.49, 6.42)	0.252
High	−13.96 (−23.48, −4.45)	0.004	−25.68 (−36.15, −15.21)	<0.001	−14.01 (−29.34, 1.33)	0.073

SUA tertiles = male T1 23.8–321.2 *μ*mol/L; T2 327.1–386.6 *μ*mol/L; T3 392.6–773.2 *μ*mol/L; Female T1 23.8–243.9 *μ*mol/L; T2 249.8–303.3 *μ*mol/L; T3 309.3–1,070.6 *μ*mol/L. LDL-c tertiles = T1 0.23–2.43 mmol/L; T2 2.46–3.15 mmol/L; T3 3.18–9.69 mmol/L; HDL-c tertiles = T1 0.16–1.14 mmol/L; T2 1.16–1.47 mmol/L; T3 1.5–5.84 mmol/L; TG tertiles = T1 0.10–0.89 mmol/L; T2 0.90–1.47 mmol/L; T3 1.48–68.38 mmol/L; and Adjusted for age, sex, race/ethnicity, BMI, glucose, smoking, alcohol consumption, SUA, LDL, TG, HDL, SBP, ALT, AST, and creatinine were adjusted. In the subgroup analysis stratified by SUA, HDL-c, LDL-c, and TG tertiles, the model was not adjusted for SUA, HDL-c, LDL-c and TG, respectively.

#### The Effect of PA on Insulin Based on LDL-c Tertiles

There was a negative correlation between LDL-c and insulin, and the level of insulin decreased as the intensity of PA improved under the same LDL-c level (Supplementary Figure S2). Table 3 shows the *β* values of insulin associated with diverse levels of PA among participants grouped based on LDL-c tertiles. Participants in the lower and upper LDL-c tertiles had a significant decrease in the level of insulin [*β* value (95% CI) = −11.16 (−19.14, −3.18), −9.90 (−18.37, −1.43) in the lower tertile, and −10.29 (−17.09, −3.49), −15.26 (−22.33, −8.18) in the upper tertile, respectively] in the general population compared to participants in the low-intensity PA group. In the middle LDL-c tertile, only the high-intensity PA group had a significant decrease in insulin, with a *β* value (95% CI) = −16.21 (−25.32, −7.10). After adjusting for potential confounders, a similar significant decrease in the level of insulin was observed among male participants in the lower and the upper tertiles. However, in females (Supplementary Table S1), the multivariate logistic regression confirmed that only participants in the upper LDL-c tertile had a significant decrease in the level of insulin, with a gradual decrease as the intensity of physical exercise increased.

#### The Effect of PA on Insulin Based on HDL-c Tertiles

There was also a negative correlation between HDL-c and insulin, and the level of insulin decreased as the intensity of PA improved under the same HDL-c level (Supplementary Figure S3). Table 3 shows the *β* value of insulin associated with an increase in PA among participants grouped by HDL-c tertiles. In the general population, a gradual decrease of *β* value in insulin was confirmed for the first HDL-c tertile [*β* value (95% CI) = −20.85 (−31.67, −10.02), and −24.19 (−35.29, −13.08)] compared to participants in the low-intensity PA group. However, in the second and third HDL-c tertiles, only participants in the high-intensity PA group had a significant decrease in the level of insulin, with *β* values (95% CI) = −10.33 (−16.96, −3.70) and −6.20 (−10.39, −2.01), respectively. Similarly, the *β* value of insulin gradually decreased across male participants in the first HDL-c tertile. Meanwhile, in second HDL-c tertile, only participants in the high-intensity PA group had a significant decrease in the level of insulin. Notably, there was no significant statistical difference in the third HDL-c tertile. In females (Supplementary Table S1), the *β* value of insulin only reduced for participants in the high-intensity PA group under the third HDL-c tertile.

#### The Effect of PA on Insulin Based on TG Tertiles

Furthermore, there was a positive correlation between TG and insulin, and the level of insulin decreased as the intensity of PA improved under the same TG level (Supplementary Figure S4). Table 3 shows the *β* value of insulin associated with an increase in PA among participants grouped based on TG tertiles. A gradual decrease in the *β* value in insulin was observed for the first and second TG tertiles [*β* value (95% CI) = −6.75 (−11.78, −1.72), −9.33 (−14.30, −4.36) and −10.18 (−17.15, −3.21), −15.38 (−22.66, −8.09), respectively] among the general population compared to the low-intensity physical group. However, in the third TG tertile, only participants in the high-intensity PA group had a significant decrease in the level of insulin, with a *β* value (95% CI) = −11.36 (−20.87, −1.84). Similarly, the *β* value of insulin gradually decreased across male participants in the first and second TG tertiles. However, there was no significant difference in the third TG tertile. In females (Supplementary Table S1), the *β* value of insulin only decreased in the high-intensity PA group under the first TG tertile.

### Sensitivity Analysis in Participants Without DM

**Figure 2:**
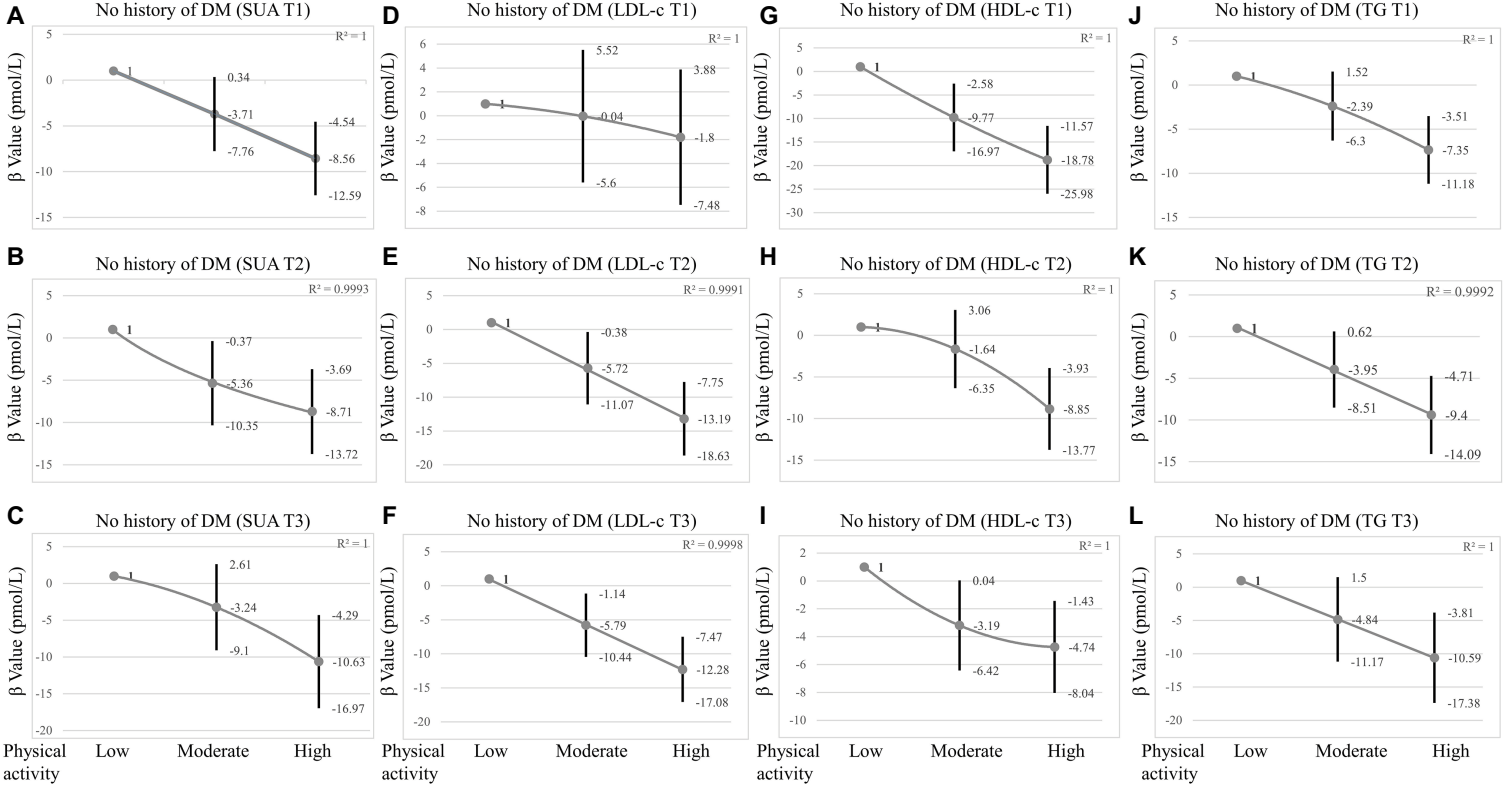
Sensitivity analysis in participants without DM grouped by SUA tertiles, LDL-c tertiles, HDL-c tertiles, and TG tertiles. **(A–C)** The association between physical activity and insulin grouped by SUA tertiles in participants without DM. **(D–F)** The association between physical activity and insulin grouped by LDL-c tertiles in participants without DM. **(G–I)** The association between physical activity and insulin grouped by HDL-c tertiles in participants without DM. **(J–L)** The association between physical activity and insulin grouped by TG tertiles in participants without DM. SUA tertiles = male T1 23.80–321.20 μmol/L; T2 327.10–380.70 μmol/L; T3 386.60–773.20 μmol/L; Female T1 23.80–243.90 μmol/L; T2 249.80–297.40 μmol/L; T3 303.30–1070.60 μmol/L. LDL-c tertiles = T1 0.23–2.46 mmol/L; T2 2.48–3.23 mmol/L; T3 3.26–9.70 mmol/L; HDL-c tertiles = T1 0.16–1.16 mmol/L; T2 1.19–1.50 mmol/L; T3 1.53–5.84 mmol/L; TG tertiles = T1 0.10–0.87 mmol/L; T2 0.88–1.40 mmol/L; T3 1.41–34.56 mmol/L; and Adjusted for age, sex, race/ethnicity, BMI, glucose, smoking, alcohol consumption, SUA, LDL-c, TG, HDL-c, SBP, ALT, AST, and creatinine were adjusted. In subgroup analysis stratified by SUA, HDL-c, LDL-c, and TG tertiles, the model is not adjusted for SUA, HDL-c, LDL-c, and TG, respectively.

It is necessary to consider DM as an important confounding factor. A sensitivity analysis was performed based on whether the participant was diagnosed with diabetes, and the relationship between PA and insulin was observed in participants without diabetes. Multivariate logistic regression showed that PA was also negatively correlated with insulin levels in participants without diabetes. The insulin levels decreased gradually with increasing intensity of PA [*β* value (95% CI) = −4.24 (−7.23, −1.25), *p* = 0.005, *β* value (95% CI) = −9.64 (−12.7, −6.58), *p* < 0.001 respectively, Table 4] after adjusting for confounders (Model II). When grouped by sex, this association persisted in males, with *β* value (95% CI) = −7.16 (−12.47, −1.84), *β* value (95% CI) = −14.63 (−19.68, −9.58), respectively. However, in females, the significant negative association only persisted in the high-intensity PA group after adjusting for confounders (Model II), with *β* value (95% CI) = −6.22 (−9.95, −2.50). When grouped by SUA tertiles, LDL-c tertiles, HDL-c tertiles, and TG tertiles, respectively, high-intensity PA significantly decreased insulin levels in three tertiles of SUA, HDL-c, TG, and in T2 and T3 of LDL-c (Figure 2 and Supplementary Table S2). Moreover, the link between PA and insulin was also stronger in males (Table 4 and Supplementary Table S2).

**Table 4:** The association between physical activity and insulin in participants without DM.

Physical activity	Crude model		Model I		Model II	
	*β* (95% CI)	*p* value	*β* (95% CI)	*p* value	*β* (95% CI)	*p* value
Low	Ref.		Ref.		Ref.	
Moderate	−9.19 (−12.82, −5.55)	<0.001	−9.46 (−13.09, −5.83)	<0.001	−4.24 (−7.23, −1.25)	0.005
High	−19.28 (−22.82, −15.74)	<0.001	−23.29 (−26.98, −19.60)	<0.001	−9.64 (−12.71, −6.58)	<0.001
**Male**						
Low	Ref.		Ref.		Ref.	
Moderate	−9.31 (−15.74, −2.89)	0.005	−9.20 (−15.64, −2.77)	0.005	−7.16 (−12.47, −1.84)	0.008
High	−22.68 (−28.63, −16.74)	<0.001	−24.71 (−30.79, −18.63)	<0.001	−14.63 (−19.68, −9.58)	<0.001
**Female**						
Low	Ref.		Ref.		Ref.	
Moderate	−9.85 (−14.08, −5.61)	<0.001	−9.66 (−13.87, −5.45)	<0.001	−3.16 (−6.56, 0.24)	0.069
High	−20.67 (−25.16, −16.19)	<0.001	−21.57 (−26.15, −16.99)	<0.001	−6.22 (−9.95, −2.50)	0.001

Model I: age, sex, and race/ethnicity were adjusted. Model II: age, sex, race/ethnicity, BMI, SBP, HDL-c, LDL-c, TG, ALT, AST, SUA, glucose, creatinine, smoking, and alcohol consumption were adjusted. DM, diabetes mellitus; Ref, reference.

## Discussion

It is well known that PA improves IR (Sampath Kumar et al., 2019). Herein, our logistic regression analyses showed that increased intensities of PA could significantly reduce insulin levels, and this tendency persisted in different stratified analysis. The link between PA and insulin persisted even after adjusting for confounding factors, independent of gender. High-intensity PA significantly lowered insulin levels in the lower and higher SUA tertiles, and in three tertiles of lipid indices (LDL-c, HDL-c, and TG) in the general population. In addition, the association between PA and insulin was stronger in male individuals than in females, and sensitivity analysis observed similar link between PA and insulin in participants without DM. Collectively, these results revealed that different intensities of PA had different effects on insulin under different lipid indices (LDL-c, HDL-c, and TG) and SUA levels. To the best of our knowledge, this is the first study to show the association between PA and insulin under different levels of SUA and lipid indices (LDL-c, HDL-c, and TG).

Insulin is the only hormone in the body that reduces blood glucose while also promoting the production of glycogen, fat, and protein. Insulin resistance occurs when the pancreas secretes a substantial amount of insulin to maintain glucose levels in the normal range. Evidence suggests that as many as 86 million Americans aged 20 and older suffer from insulin resistance (National Center for Chronic Disease and Health Promotion, 2014). In modern cultures, noncommunicable diseases, such as CVD, diabetes, and obesity, which account for more than 70% of all deaths worldwide, are on the rise (World Health Organization, 2018). Despite the high costs of chronic diseases, it is expected that the majority of noncommunicable diseases can be avoided. Physical inactivity is a big risk factor, and hence, PA is an obvious remedy, in addition to a poor diet, cigarette use, and problematic alcohol consumption. For example, it has been found that physically inactive middle-aged women have a 52% increases in all-cause death and a doubling in cardiovascular-related mortality (Hu et al., 2004; Myers et al., 2004). MS risk decreases have been recorded with as little as 30 min of moderate-intensity activity per day, and the process involves numerous pathways, including body mass regulation, hypertension, insulin resistance reduction, dyslipidemia, insulin sensitivity enhancement, and glycemic control (Bassuk and Manson, 2005).

This study found that increased intensities of PA can considerably reduce insulin levels, with high-intensity PA exhibiting the best results. The American College of Sports Medicine suggests 30–60 min of moderate-intensity aerobic exercise 5 or more days per week, or three or more 20–60 min vigorous-intensity exercise sessions (Garber et al., 2011). Although moderate-intensity exercise is beneficial in this regard, some studies have revealed that strenuous exercise is even more effective (Slentz et al., 2005; DiPietro et al., 2006; Swain and Franklin, 2006). The odds ratios for having MS in the Whitehall II research, which included 5,153 Caucasian Europeans, were 0.52 and 0.78, respectively, among persons engaging in high (METs > 5) and moderate (3 < METs < 5) activity (Rennie et al., 2003). These recommendations are consistent with mounting evidence that high-intensity training can be just as effective as traditional high-volume endurance training at moderate intensities, not only in terms of endurance performance improvements, but also in terms of health benefits, with some studies even indicating that high-intensity training may be superior (Wisloff et al., 2009; Kessler et al., 2012). The above recommendations, undoubtedly, better support our results.

This study confirmed that SUA and insulin were positively correlated. The strong intercorrelation between hyperuricemia and IR has been well demonstrated in previous studies. Some studies have reported that increased uric acid levels can predict the risk of IR (Krishnan et al., 2012) and higher uric acid levels probably precede insulin resistance (Han et al., 2017). It has been reported that lower uric acid levels with allopurinol can improve IR (Nakagawa et al., 2006; Takir et al., 2015). In this study, results obtained in the lower and higher SUA tertiles also showed that high-intensity PA could significantly reduce insulin levels. The underlying mechanism of this association may be clarified from the aspect of redox in the body. It is well known that SUA has a physiological function, acting as an antioxidant by enhancing superoxide dismutation to hydrogen peroxide and lowering superoxide availability and its detrimental interaction with nitric oxide (Davies et al., 1986). When the level of uric acid gradually rises, it will produce pro-oxidant properties. Hepatic IR can be caused by high uric acid levels, which cause hepatic steatosis by causing mitochondrial oxidative stress (Lanaspa et al., 2012). Elevated uric acid can cause peripheral IR through two main mechanisms: (1) decreased NO bioavailability and endothelial NO supply, which restricts glucose delivery to skeletal muscle (Roy et al., 1998; Khosla et al., 2005) and (2) activation of NADPH oxidase, which produces oxidized lipids and inflammatory mediators in adipocytes (Sautin et al., 2007). In addition, participants with high SUA seem to have an unhealthy lifestyle (Hu et al., 2020). In a recent investigation, SUA levels were found to be favorably linked with all indices of adiposity (Pirro et al., 2017). Similarly, another study found that adiposity variables could partially or completely mediate the relationship between SUA, glucose/insulin homeostasis, and inflammation (Mazidi et al., 2018). However, low SUA levels might reflect persons with a poor nutritional status (Beberashvili et al., 2015). Therefore, low SUA levels represent reduced total antioxidant capacity. Regular aerobic exercise improves antioxidant defenses and immunological response, which helps to improve vascular and cellular health (He et al., 2016). Furthermore, the positive effects of daily PA on oxidative stress levels have been demonstrated in patients with atherosclerosis (Gardner et al., 2017). To reduce oxidative damage, cells increase *de novo* synthesis of antioxidant enzymes during persistent exercise training. SOD has been shown to rise in response to exercise training (Toledo-Arruda et al., 2017), as one of the first antioxidant enzymes to respond to cellular ROS. Chronic PA has also been demonstrated to boost the two other primary antioxidant enzymes, glutathione peroxidase and catalase (Rowinski et al., 2013; Fraile-Bermudez et al., 2015). These results obtained in this study suggested that high-intensity PA still reduced insulin levels under conditions of oxidative stress of the body, possibly because PA can not only reduce weight, but also stabilize oxidative stress levels in the body, thereby increasing insulin sensitivity and reducing insulin levels. It is well known that both insulin resistance and insulin secretion defects are two core mechanisms during the development of DM. A series of cohort studies and a subsequent meta-analysis investigated the relationship between SUA levels and the incidence of impaired fasting glucose (IFG), and T2DM and discovered that hyperuricemia is an early and important sign of impaired glucose control (Krishnan et al., 2012; Jia et al., 2013; Cicero et al., 2014). Therefore, sensitivity analysis was performed in participants without DM. Interestingly, we observed that high-intensity PA reduced insulin levels at all levels of SUA. This may be related to the antioxidant of SUA itself and the complicated relationship between SUA and IR and DM, but the specific mechanism needs further epidemiological research and basic experimental studies to confirm.

In this study, we found a positive correlation between TG and insulin, and a negative correlation between LDL-c, HDL-c, and insulin. It is widely recognized that insulin resistance (IR) plays a critical role in the pathogenesis of dyslipidemia. However, in contrast, one study suggested that lipid buildup also causes IR (Medina-Santillan et al., 2013). Studies have shown that IR impacts the metabolism of triglycerides, HDL-c, and low-density lipoprotein cholesterol (LDL-c) through several mechanisms (Grundy, 1997; Festa et al., 2005; Bjornstad and Eckel, 2018). Increased levels of hepatic triglyceride lipase (HTGL) have also been associated with IR, which may result in faster HDL-c clearance and lower HDL-c levels (Baynes et al., 1991; Sparks et al., 2012), ultimately causing hypertriglyceridemia and reduced HDL-c values. It should be noted that IR and dyslipidemia are risk factors for CVDs and DM. Recent research on the relationship between physical inactivity and CVD has yielded sobering results, showing that physical inactivity is a potential risk factor that considerably increases susceptibility to CVD (Erlichman et al., 2002). In an RCT study, which the overall effects of PA were analyzed by quartiles of daily steps of all subjects, there were significant reductions in total and LDL cholesterol and visceral fat area between the highest (daily steps over 6,520) and the lowest quartile (1780–2,810 daily steps) and they confirmed that habitual and structured PA with the acceleration levels of 0.3–0.7 g and daily steps over 6,520 are clinically beneficial for overweight, obese, and physically inactive individuals with a high risk for T2DM (Herzig et al., 2014). In a variety of populations, including men and women with diabetes, glucose intolerance, obesity, sedentary moderately overweight, metabolic syndrome, and T2DM, recent studies have consistently shown that moderate aerobic exercise for 30 min or more three times per week for at least 8 weeks improves insulin sensitivity (Bird and Hawley, 2016). Furthermore, PA has been used as a therapeutic strategy for the prevention of CVD and DM (Pearson et al., 2002). Previous studies have focused on that PA not only improves IR, but also improves lipid homeostasis (Herzig et al., 2014) and body weight (Tuomilehto et al., 2001), reducing the risk of T2DM. In our study, however, the high-intensity PA effects on insulin were statistically significant regardless of changes in lipid indices (TG, HDL-c, and LDL-c) levels and other confounding factors. The improvement in the insulin levels of our participants appeared to be mostly an independent outcome and is not affected by lipid levels. Our new results in sensitivity analysis also confirmed that the change in PA had an independent effect on insulin levels regardless of the levels of lipid indices (TG, HDL-c, and LDL-c) in participants without DM, and these results may reflect the effects of PA on insulin signaling in the skeletal muscle (Despres et al., 2001).

Interestingly, we found that the relationship between PA and insulin was more pronounced in men. According to numerous research conducted predominantly in male populations (Lehtonen and Viikari, 1978; Huttunen et al., 1979), increased intensities of PA are associated with higher HDL cholesterol levels and lower triglyceride levels. Recent studies have revealed that sex hormones may play a role in the control of insulin receptors (Bertoli et al., 1980) and the maintenance of beta-cell competence (Bailey and Ahmed-Sorour, 1980). The lack of significant results in women suggests that estrogen and/or progesterone may influence the relationship between insulin sensitivity, PA, and lipoprotein indices. In addition, there are gender differences in substrate utilization during exercise (Ruby and Robergs, 1994). However, the role of sex hormones in this pathway is unclear, and thus further methodological studies should be conducted.

However, the study had some limitations. To begin with, the cross-sectional study design did not rule out the possibility of a causal link between SUA, lipid, and insulin levels. Second, we were unable to rule out the impact of underlying disorders and medications, particularly hypoglycemic medicines, on the outcomes. Further basic mechanism research and a large population-based sample should be conducted in a prospective manner to solve these constraints.

## Conclusion

In conclusion, this study shows that PA can significantly lower insulin levels, and high-intensity PA still has additional potential benefits for insulin levels, even in the condition of dyslipidemia and hyperuricemia. When properly programmed, regular PA can not only reduce risk factors for a range of noncommunicable diseases, such as CVD, sarcopenia, metabolic syndrome, osteoporosis, and depression, but also increase physical performance (strength, power, and endurance), physical, and mental health. Unlike medication, PA typically has no adverse effects, is inexpensive, cures multiple health concerns at once, and may have extra potential advantages.

## Data Availability Statement

The datasets presented in this study can be found in online repositories. The names of the repository/repositories and accession number(s) can be found in the article/Supplementary Material.

## Author Contributions

YL and RF: conceptualization. YL, RF, ZH, and JL: methodology. YX: validation, resources, and project administration. YL, RF, and JL: formal analysis. YX and XY: investigation. YL, RF, and ZH: writing – original draft preparation. YX, YZ, and XY: writing – review and editing. YX and YZ: funding acquisition. All authors contributed to the article and approved the submitted version.

## Funding

This research was funded by the National Natural Science Foundation of China (grant numbers 81900439 and 81970286), the Major Program of Science and Technology of Liaoning (2021JH1/10400050), the Science Foundation of Doctors of Liaoning Province (grant number 2020-BS-197), the Chang Jiang Scholars Program (grant number T2017124), and the Dalian Talents Innovation Supporting Project (grant number 2018RD09).

## Conflict of Interest

The authors declare that the research was conducted in the absence of any commercial or financial relationships that could be construed as a potential conflict of interest.

## Publisher’s Note

All claims expressed in this article are solely those of the authors and do not necessarily represent those of their affiliated organizations, or those of the publisher, the editors and the reviewers. Any product that may be evaluated in this article, or claim that may be made by its manufacturer, is not guaranteed or endorsed by the publisher.

## Footnotes

^1^ http://www.cdc.gov/nchs/nhanes/

^2^ http://www.empowerstats.com

^3^ http://www.R-project.org/
