# Supplementary material for: The Association Between Physical Activity and Insulin Level Under Different Levels of Lipid Indices and Serum Uric Acid
Source: Front Physiol. 2022 Feb 2;13:809669. doi: 10.3389/fphys.2022.809669 (PMC8847671; doi:10.3389/fphys.2022.809669)
Supplement: Supplementary file 1 [file Data_Sheet_1.docx]

**Supplementary material**

**Table S1 The association between physical activity and insulin grouped by SUA/LDL-c/HDL-c/TG tertiles in females**

| Physical activity | Tertile 1 of SUA | | Tertile 2 of SUA | | Tertile 3 of SUA | |
| --- | --- | --- | --- | --- | --- | --- |
|  | β (95%CI) | P value | β (95%CI) | P value | β (95%CI) | P value |
| Low | Ref. |  | Ref. |  | Ref. |  |
| Moderate | -5.23 (-10.60, 0.15) | 0.057 | -3.11 (-12.03, 5.80) | 0.494 | -5.38 (-13.55, 2.79) | 0.197 |
| High | **-6.18 (-11.88, -0.47)** | **0.034** | -4.46 (-14.06, 5.14) | 0.362 | -9.96 (-20.11, 0.20) | 0.055 |
| Physical activity | Tertile 1 of LDL-c | | Tertile 2 of LDL-c | | Tertile 3 of LDL-c | |
|  | β (95%CI) | P value | β (95%CI) | P value | β (95%CI) | P value |
| Low | Ref. |  | Ref. |  | Ref. |  |
| Moderate | -0.93 (-8.81, 6.95) | 0.817 | -7.83 (-17.68, 2.01) | 0.119 | **-7.87 (-13.93, -1.81)** | **0.011** |
| High | 0.28 (-8.38, 8.95) | 0.949 | -8.80 (-19.79, 2.19) | 0.117 | **-13.56 (-20.50, -6.62)** | **<0.001** |
| Physical activity | Tertile 1 of HDL-c | | Tertile 2 of HDL-c | | Tertile 3 of HDL-c | |
|  | β (95%CI) | P value | β (95%CI) | P value | β (95%CI) | P value |
| Low | Ref. |  | Ref. |  | Ref. |  |
| Moderate | -7.28 (-20.94, 6.38) | 0.296 | -4.62 (-13.51, 4.28) | 0.309 | -4.12 (-8.63, 0.39) | 0.074 |
| High | -8.34 (-24.58, 7.89) | 0.314 | -8.39 (-18.77, 1.99) | 0.113 | **-5.96 (-10.80, -1.12)** | **0.016** |
| Physical activity | Tertile 1 of TG | | Tertile 2 of TG | | Tertile 3 of TG | |
|  | β(95%CI) | P value | β(95%CI) | P value | β(95%CI) | P value |
| Low | Ref. |  | Ref. |  | Ref. |  |
| Moderate | -4.90 (-10.09, 0.29) | 0.064 | -3.28 (-12.38, 5.81) | 0.479 | -5.59 (-14.10, 2.92) | 0.198 |
| High | **-7.15 (-12.48, -1.82)** | **0.008** | -7.19 (-17.48, 3.11) | 0.172 | -3.82 (-14.62, 6.97) | 0.488 |

SUA tertiles= male T1 23.80-321.20 μmol/L; T2 327.10-386.60 μmol/L; T3 392.60-773.20 μmol/L; Female T1 23.80-243.90 μmol/L; T2 249.80-303.30 μmol/L; T3 309.30-1070.60 μmol/L. LDL-c tertiles= T1 0.23-2.43 mmol/L; T2 2.46-3.15 mmol/L; T3 3.18-9.69 mmol/L; HDL-c tertiles= T1 0.16-1.14 mmol/L; T2 1.16-1.47 mmol/L; T3 1.5-5.84 mmol/L; TG tertiles= T1 0.10-0.89 mmol/L; T2 0.90-1.47 mmol/L; T3 1.48-68.38 mmol/L; Adjusted for age, sex, race/ ethnicity, BMI, glucose, smoking, alcohol consumption, SUA, LDL-c, TG, HDL-c, SBP, ALT, AST, creatinine, were adjusted. In the subgroup analysis stratified by SUA, HDL-c, LDL-c, and TG tertiles, the model is not adjusted for SUA, HDL-c, LDL-c and TG, respectively.

**
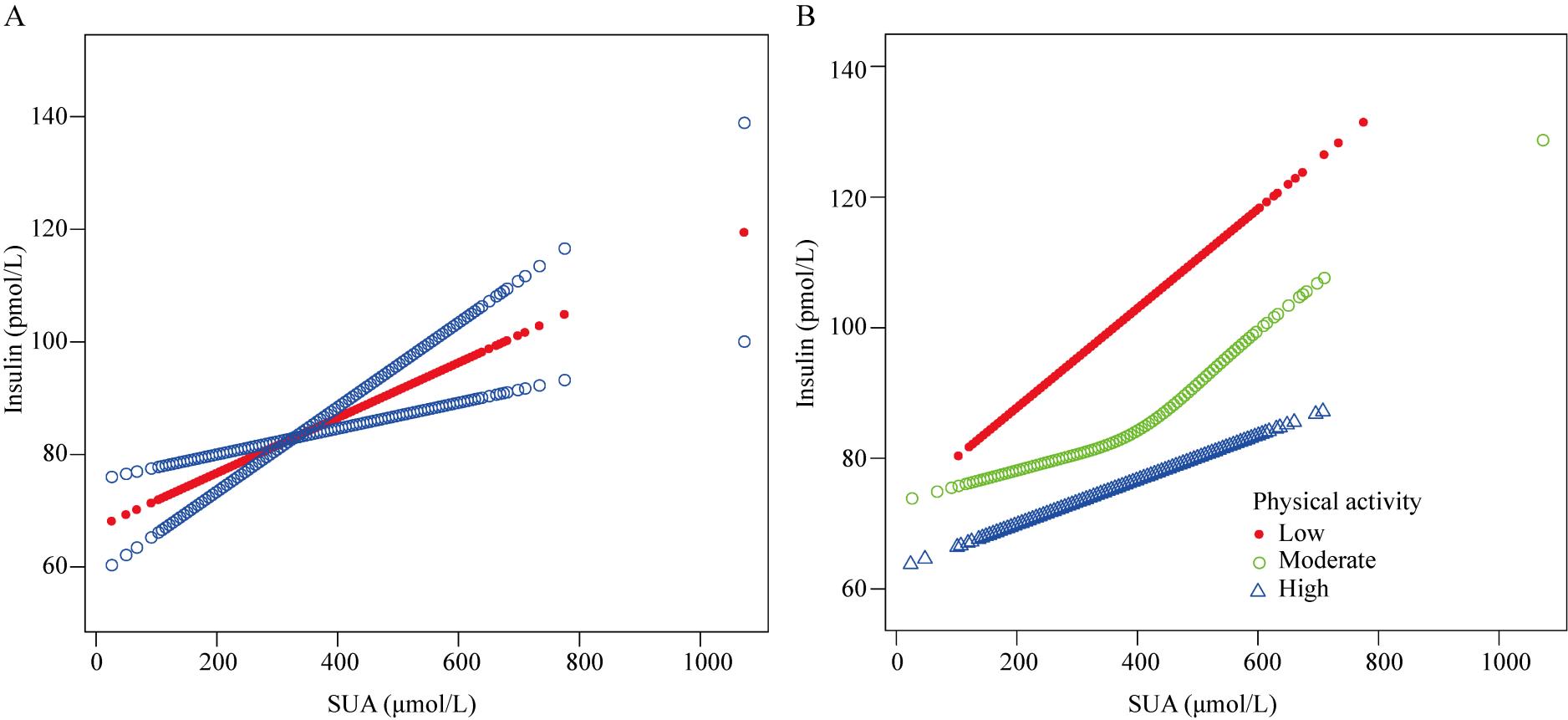
**

**Figure S1 The association between serum uric acid and insulin.**

1. Solid rad line represents the smooth curve fit between variables. Blue bands represent the 95% of confifidence interval from the fit. Age, sex, race/ ethnicity, BMI, glucose, smoking, alcohol consumption, LDL-c, TG, HD-cL, SBP, ALT, AST, creatinine, physical activity, were adjusted. (B) The association between serum uric acid and insulin grouped by the leves of physical activity. Age, sex, race/ ethnicity, BMI, glucose, smoking, alcohol consumption, LDL-c, TG, HDL-c, SBP, ALT, AST, creatinine, were adjusted.


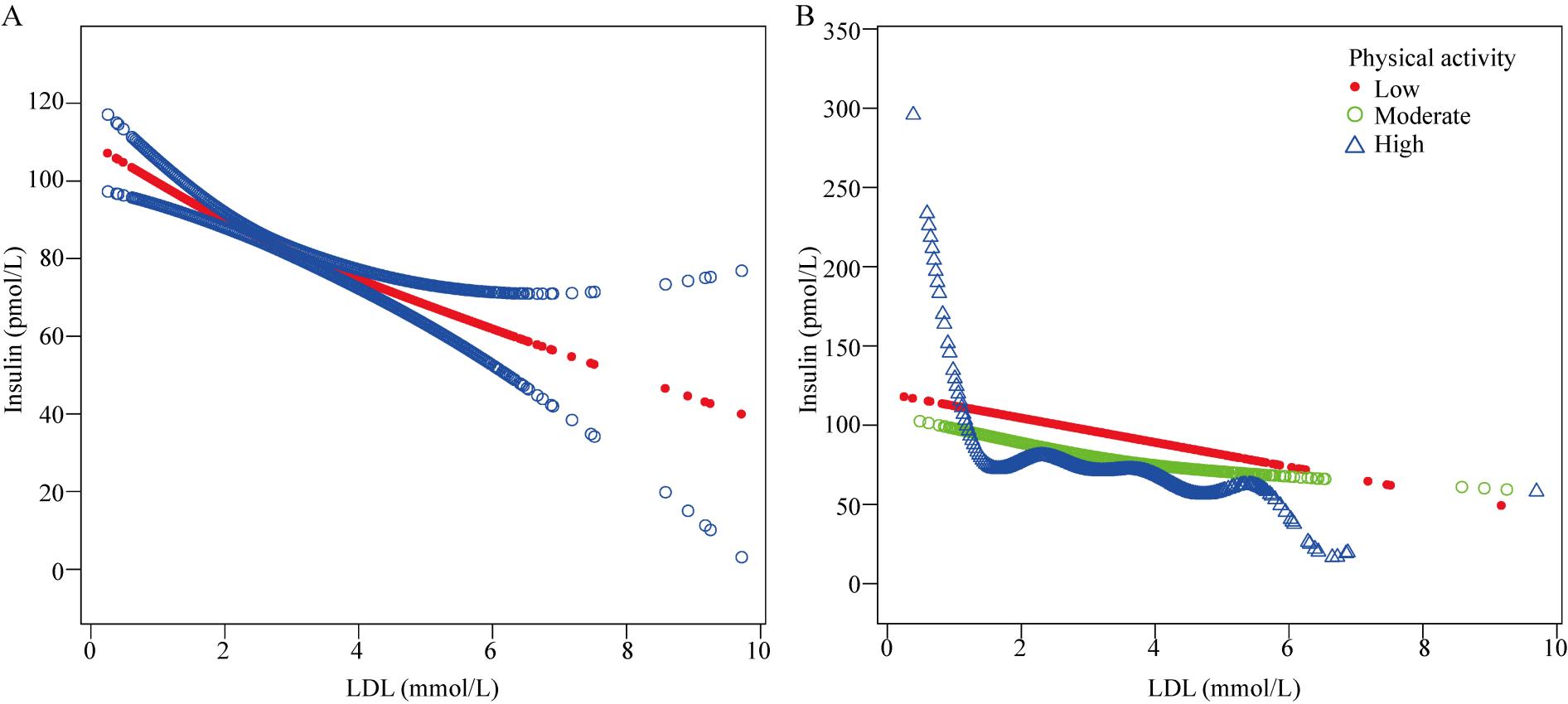


**Figure S2 The association between LDL-c and insulin.**

(A) Solid rad line represents the smooth curve fit between variables. Blue bands represent the 95% of confifidence interval from the fit. Age, sex, race/ ethnicity, BMI, glucose, smoking, alcohol consumption, SUA, TG, HDL-c, SBP, ALT, AST, creatinine, physical activity, were adjusted. (B) The association between LDL-c and insulin grouped by the leves of physical activity. Age, sex, race/ ethnicity, BMI, glucose, smoking, alcohol consumption, SUA, TG, HDL-c, SBP, ALT, AST, creatinine, were adjusted.


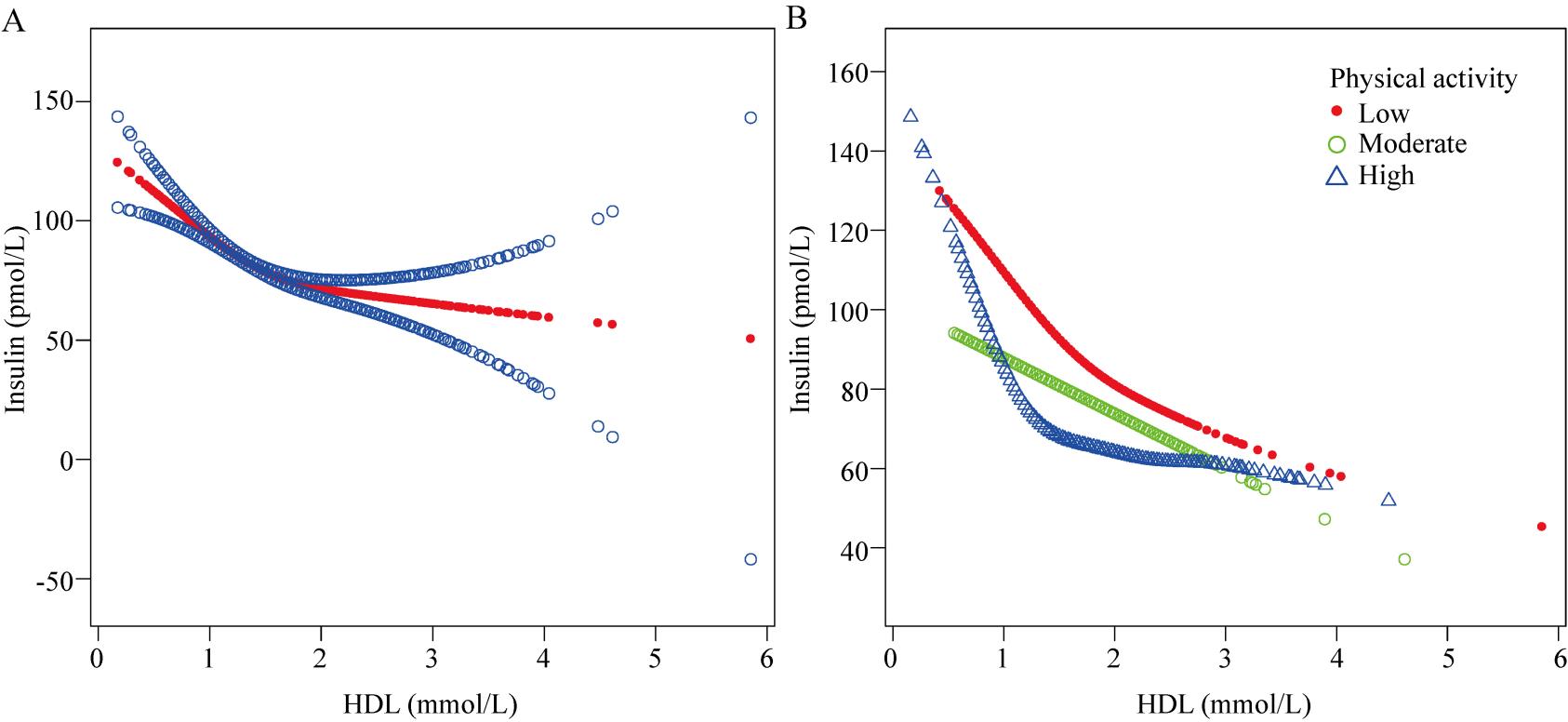


**Figure S3 The association between HDL-c and insulin.**

1. Solid rad line represents the smooth curve fit between variables. Blue bands represent the 95% of confifidence interval from the fit. Age, sex, race/ ethnicity, BMI, glucose, smoking, alcohol consumption, SUA, TG, LDL-c, SBP, ALT, AST, creatinine, physical activity, were adjusted. (B) The association between HDL and insulin grouped by the leves of physical activity. Age, sex, race/ ethnicity, BMI, glucose, smoking, alcohol consumption, SUA, TG, LDL-c, SBP, ALT, AST, creatinine, were adjusted.

**
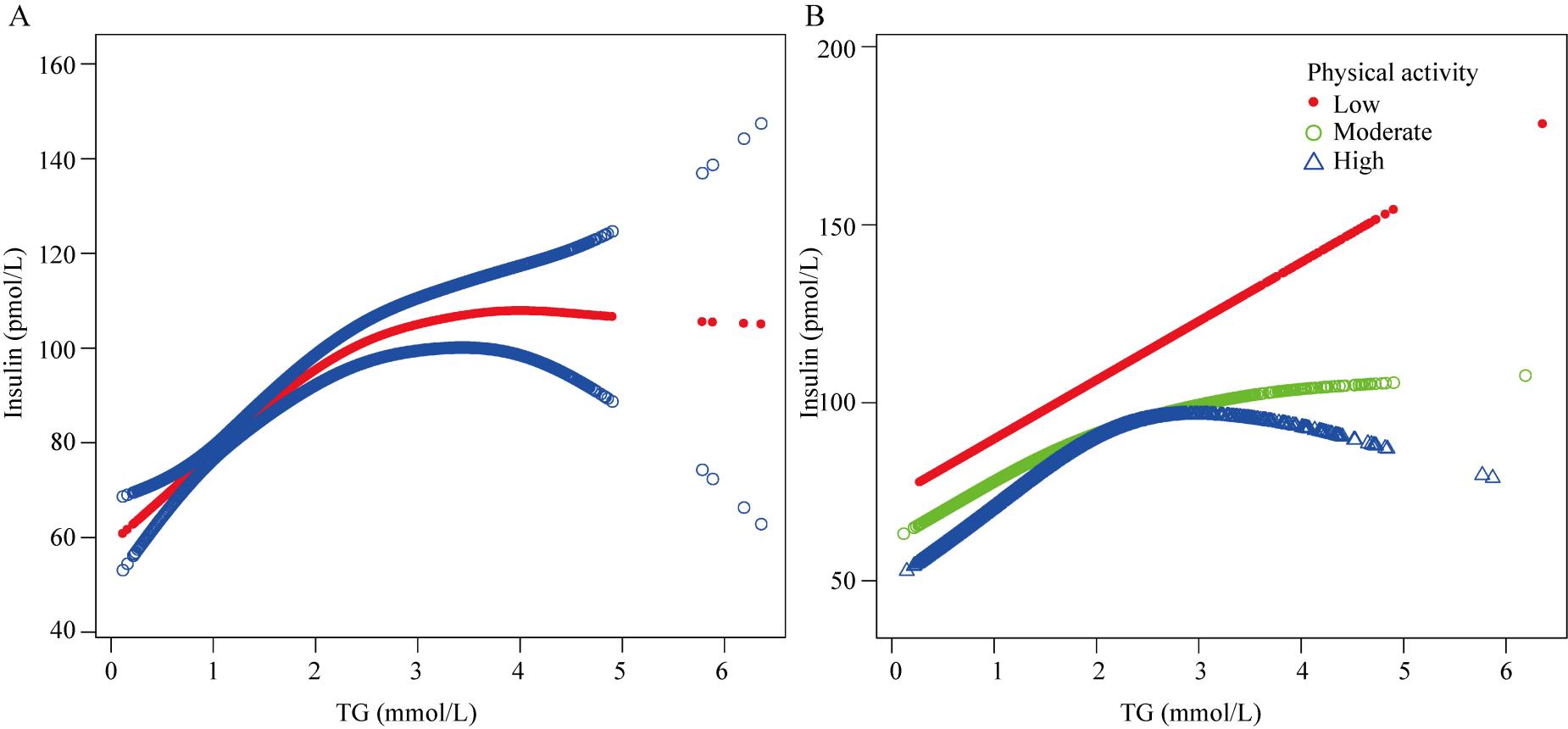
**

**Figure S4** **The association between TG and insulin.**

(A) Solid rad line represents the smooth curve fit between variables. Blue bands represent the 95% of confifidence interval from the fit. Age, sex, race/ ethnicity, BMI, glucose, smoking, alcohol consumption, SUA, LDL-c, HDL-c, SBP, ALT, AST, creatinine, physical activity, were adjusted. (B) The association between TG and insulin grouped by the leves of physical activity. Age, sex, race/ ethnicity, BMI, glucose, smoking, alcohol consumption, SUA, LDL-c, HDL-c, SBP, ALT, AST, creatinine, were adjusted.

**Table S2 The association between physical activity and insulin grouped by SUA, LDL-c, HDL-c, TG tertiles in participants without DM.**

| Physical activity | Tertile 1 of SUA | | Tertile 2 of SUA | | Tertile 3 of SUA | |
| --- | --- | --- | --- | --- | --- | --- |
|  | β (95%CI) | P value | β (95%CI) | P value | β (95%CI) | P value |
| Low | Ref. |  | Ref. |  | Ref. |  |
| Moderate | -3.71 (-7.76, 0.34) | 0.073 | **-5.36 (-10.35, -0.37)** | **0.035** | -3.24 (-9.10, 2.61) | 0.278 |
| High | **-8.56 (-12.59, -4.54)** | **<0.001** | **-8.71 (-13.72, -3.69)** | **<0.001** | **-10.63 (-16.97, -4.29)** | **0.001** |
| Male | | | | | | |
| Low | Ref. |  | Ref. |  | Ref. |  |
| Moderate | -5.12 (-12.66, 2.42) | 0.183 | -7.06 (-15.42, 1.31) | 0.098 | -8.50 (-19.23, 2.23) | 0.121 |
| High | **-13.71 (-20.73, -6.68)** | **<0.001** | **-13.21 (-21.17, -5.25)** | **0.001** | **-12.77 (-23.17, -2.38)** | **0.016** |
| Female | | | | | | |
| Low | Ref. |  | Ref. |  | Ref. |  |
| Moderate | -4.45 (-8.98, 0.08) | 0.054 | -4.65 (-10.50, 1.20) | 0.120 | -1.14 (-7.73, 5.44) | 0.734 |
| High | **-5.34 (-10.07, -0.61)** | **0.027** | -5.20 (-11.41, 1.00) | 0.100 | **-10.28 (-18.18, -2.38)** | **0.011** |
| Physical activity | Tertile 1 of LDL-c | | Tertile 2 of LDL-c | | Tertile 3 of LDL-c | |
|  | β (95%CI) | P value | β (95%CI) | P value | β (95%CI) | P value |
| Low | Ref. |  | Ref. |  | Ref. |  |
| Moderate | -0.04 (-5.60, 5.52) | 0.989 | **-5.72 (-11.07, -0.38)** | **0.036** | **-5.79 (-10.44, -1.14)** | **0.015** |
| High | -1.80 (-7.48, 3.88) | 0.534 | **-13.19 (-18.63, -7.75)** | **<0.001** | **-12.28 (-17.08, -7.47)** | **<0.001** |
| Male | | | | | | |
| Low | Ref. |  | Ref. |  | Ref. |  |
| Moderate | -4.76 (-15.12, 5.60) | 0.368 | -4.17 (-13.51, 5.18) | 0.383 | **-12.55 (-20.56, -4.54)** | **0.002** |
| High | -6.98 (-17.17, 3.22) | 0.180 | **-16.89 (-25.60, -8.18)** | **<0.001** | **-19.12 (-26.62, -11.61)** | **<0.001** |
| Female | | | | | | |
| Low |  |  |  |  |  |  |
| Moderate | 3.23 (-2.80, 9.26) | 0.294 | -8.45 (-14.64, -2.26) | 0.008 | -2.70 (-8.16, 2.76) | 0.333 |
| High | 1.42 (-4.92, 7.75) | 0.662 | **-10.37 (-17.17, -3.56)** | **0.003** | **-7.43 (-13.62, -1.25)** | **0.019** |
| Physical activity | Tertile 1 of HDL-c | | Tertile 2 of HDL-c | | Tertile 3 of HDL-c | |
|  | β (95%CI) | P value | β (95%CI) | P value | β (95%CI) | P value |
| Low | Ref. |  | Ref. |  | Ref. |  |
| Moderate | **-9.77 (-16.97, -2.58)** | **0.008** | -1.64 (-6.35, 3.06) | 0.493 | -3.19 (-6.42, 0.04) | 0.053 |
| High | **-18.78 (-25.98, -11.57)** | **<0.001** | **-8.85 (-13.77, -3.93)** | **<0.001** | **-4.74 (-8.04, -1.43)** | **0.005** |
| Male | | | | | | |
| Low | **Ref.** |  | Ref. |  | Ref. |  |
| Moderate | **-12.55 (-21.92, -3.18)** | **0.009** | 0.98 (-6.71, 8.68) | 0.802 | -6.17 (-13.79, 1.46) | 0.114 |
| High | **-21.83 (-30.71, -12.96)** | **<0.001** | **-8.66 (-16.03, -1.29)** | **0.021** | -5.43 (-12.65, 1.80) | 0.141 |
| Female | | | | | | |
| Low | Ref. |  | Ref. |  | Ref. |  |
| Moderate | -5.00 (-16.03, 6.03) | 0.374 | -3.30 (-9.36, 2.76) | 0.286 | -2.98 (-6.50, 0.55) | 0.098 |
| High | -10.50 (-23.17, 2.17) | 0.105 | **-7.61 (-14.52, -0.70)** | **0.031** | **-4.54 (-8.25, -0.84)** | **0.016** |
| Physical activity | Tertile 1 of TG | | Tertile 2 of TG | | Tertile 3 of TG | |
|  | β(95%CI) | P value | β(95%CI) | P value | β(95%CI) | P value |
| Low | Ref. |  | Ref. |  | Ref. |  |
| Moderate | -2.39 (-6.30, 1.52) | 0.232 | -3.95 (-8.51, 0.62) | 0.090 | -4.84 (-11.17, 1.50) | 0.135 |
| High | **-7.35 (-11.18, -3.51)** | **<0.001** | **-9.40 (-14.09, -4.71)** | **<0.001** | **-10.59 (-17.38, -3.81)** | **0.002** |
| Male | | | | | | |
| Low | Ref. |  | Ref. |  | Ref. |  |
| Moderate | 0.45 (-7.00, 7.90) | 0.906 | -3.46 (-11.11, 4.20) | 0.376 | **-12.63 (-23.17, -2.10)** | **0.019** |
| High | **-6.88 (-13.78, 0.03)** | **0.051** | **-9.74 (-16.99, -2.49)** | **0.009** | **-21.20 (-31.45, -10.95)** | **<0.001** |
| Female | | | | | | |
| Low | Ref. |  | Ref. |  | Ref. |  |
| Moderate | -3.93 (-8.47, 0.60) | 0.089 | -5.29 (-10.88, 0.31) | 0.064 | -0.99 (-8.36, 6.37) | 0.792 |
| High | **-7.76 (-12.37, -3.14)** | **0.001** | **-10.67 (-16.88, -4.46)** | **<0.001** | 1.92 (-7.01, 10.86) | 0.673 |

SUA tertiles= male T1 23.80-321.20 μmol/L; T2 327.10-380.70 μmol/L; T3 386.60-773.20μmol/L; Female T1 23.80-243.90 μmol/L; T2 249.80-297.40 μmol/L; T3 303.30-1070.60 μmol/L. LDL-c tertiles= T1 0.23-2.46 mmol/L; T2 2.48-3.23 mmol/L; T3 3.26-9.70 mmol/L; HDL-c tertiles= T1 0.16-1.16 mmol/L; T2 1.19-1.50 mmol/L; T3 1.53-5.84 mmol/L; TG tertiles= T1 0.10-0.87 mmol/L; T2 0.88-1.40 mmol/L; T3 1.41-34.56 mmol/L; Adjusted for age, sex, race/ ethnicity, BMI, glucose, smoking, alcohol consumption, SUA, LDL-c, TG, HDL-c, SBP, ALT, AST, creatinine, were adjusted. In the subgroup analysis stratified by SUA, HDL-c, LDL-c, and TG tertiles, the model is not adjusted for SUA, HDL-c, LDL-c and TG, respectively.
